# Scoping Review on Play-Based Interventions in Autism Spectrum Disorder

**DOI:** 10.3390/children9091355

**Published:** 2022-09-05

**Authors:** Lucía López-Nieto, Laura María Compañ-Gabucio, Laura Torres-Collado, Manuela Garcia-de la Hera

**Affiliations:** 1 Nutritional Epidemiology Unit, University of Miguel Hernández, 03550 Alicante, Spain; 2 Alicante Institute for Health and Biomedical Research (ISABIAL), 03010 Alicante, Spain; 3 Spanish Consortium for Research on Epidemiology and Public Health (CIBERESP), Instituto de Salud Carlos III, 28034 Madrid, Spain

**Keywords:** autism spectrum disorder, play-based therapy, children, adolescents, rehabilitation

## Abstract

Play as a therapeutic strategy can help to improve daily functioning in children and adolescents with autism spectrum disorder (ASD). Play-based intervention can thus be an optimal option for treatment of this population. Our aim was to describe play-based interventions used in children and adolescents with ASD. We conducted a scoping review. A peer-reviewed literature search was conducted on PubMed, Scopus, EMBASE, Web of Science and PsycINFO databases. We included experimental studies which analyzed play-based interventions in children and adolescents with ASD, which were published in English/Spanish with full text available. We used three tables elaborated a priori to perform data extraction by two authors. Fifteen studies were included, mostly conducted in Australia and published during the past 10 years. Play-based intervention was categorized into three groups: new technologies, symbolic play or official techniques. Interventions lasted approximately 11 weeks, contained one weekly session of 30–60 min and were delivered by parents and teachers. Play-based interventions using new technologies were the most used. Intervention duration and number of sessions varied between articles. Further studies are needed to create play-based intervention protocols which can be implemented in clinical practice with children and adolescents with ASD, thus promoting evidence-based interventions in this field.

## 1. Introduction

Autism spectrum disorder (ASD) is a group of neurodevelopmental disorders characterized by restrictions in social interaction, alterations in communication, a lack of cognitive flexibility, the presence of restrictive interests and stereotyped behaviors [1]. The cause of ASD is unknown, although epigenetic and environmental agents have been described as etiological factors [2]. These disorders are diagnosed four times more often in boys than girls [3], usually at nearly 4 years old [4]. The worldwide prevalence of ASD is approximately 1 in every 160 children and adolescents [5,6], although some studies have reported prevalence of up to 1 in every 138 [7,8].

ASD symptomatology affects the occupations of children and adolescents with these disorders, limiting their autonomy and independence [9]. Children and adolescents with ASD have difficulties initiating and maintaining interactions with peers, especially during play and social activities [10]. Play is the most important occupation for all children since it helps them to develop the necessary skills for the autonomous performance of daily life activities (ADL) [11]. However, the ability to play naturally is limited in children with ASD due to communication difficulties, alterations in social relationships and restricted interests [12]. This can alter their development, leading to disruption in the performance of ADL in adolescence, and even in adulthood [11]. Therefore, early detection of ASD and early interventions are essential to minimize limitations in these children’s future daily life [13].

A well-known early approach is play-based intervention, which is defined as the use of games, whether virtual or physical, in rehabilitative treatments carried out by a trained professional [14]. These interventions seek to enable children or adolescents to express themselves through play, thus increasing their motivation, patient-therapist bond and adherence to treatment [14,15]. Play-based interventions are usually led by trained adults and/or involve the participation of peers [12]. The experience of play has been related to different benefits in children and adolescents with ASD such as increased attention, exploration of the environment and self-regulation [16]. Schottelkorb et al. conducted a randomized intervention study based on self-directed play sessions, showing that children aged 4–10 years with ASD who received the intervention experienced a reduction in ASD behavioral symptoms such as attention problems and aggression compared to children in the waiting list group [15]. Similarly, another recent study has shown that a parent-led play-based intervention for children with ASD improves language and interaction at 1–4 years of age [17].

ASD has an increasing prevalence worldwide and causes limitations in the ADL performance of children and adolescents who suffer from it, as well as high levels of stress in their families [18,19,20]. Play-based interventions seem to be ideal for treating children and adolescents with ASD, since the means of intervention is play, the main occupation in this vital stage of life. Very recently, a systematic review has been published on the effectiveness of play-based interventions in children aged 1–11 years with ASD, showing a significant benefit in aspects such as social interaction, communication, daily functioning and parent–child bonding [21]. However, we have not found any review studies that summarize the characteristics of play-based interventions in this population. Therefore, we conducted a scoping review to complement the abovementioned review by answering the following research question: What are the main characteristics of play-based interventions used in children and adolescents with ASD according to the published scientific literature? Our aim was to describe play-based interventions used in children and adolescents with ASD according to the published scientific literature. The present scoping review will provide researchers interested in this topic with an up-to-date source of information that they can use in their clinical practice.

## 2. Materials and Methods

This scoping review was elaborated following the standards of the *Cochrane Handbook* Version 6.2, 2021 [22] and the PRISMA recommendations for scoping reviews (PRISMA-ScR) [23].

### 2.1. Search Strategy

On 9 November 2021, a peer-reviewed literature search was carried out in the following databases: PubMed, Scopus, EMBASE, Web of Science (WOS) and PsycINFO. The same search strategy was used in each of the abovementioned databases. Search terms included the disorders included in ASD, “game-based” and “play-based” combined with the Boolean operators “AND” and “OR”. The complete search strategies can be found in Table 1.

### 2.2. Inclusion and Exclusion Criteria

We established the following inclusion criteria: (a) experimental studies with at least 10 participants: randomized and non-randomized clinical trials, pilot studies, exploratory studies and quasi-experimental studies; (b) child or adolescent population (under 18 years old); (c) population diagnosed with ASD: Asperger’s syndrome, Rett syndrome, disintegrative disorder, classic autistic disorder and pervasive developmental disorder; and (d) studies using play-based intervention. We also established the following exclusion criteria: (a) studies published in a language other than English and/or Spanish; and (b) studies with no full text available.

We did not apply any filters by time or study type in any of the databases consulted. All inclusion and exclusion criteria were applied manually.

### 2.3. Study Selection

The review and screening of the articles identified in the literature searches of the five databases was carried out using Microsoft Excel. We downloaded all article titles from each database onto an Excel sheet, from which duplicates were removed. Once duplicates were removed, we performed the screening of the remaining studies in three phases: by title, by abstract and by full text. One researcher (LCLN) created the database in Excel and removed duplicates, two researchers (LCLN and LMCG) performed all screenings independently, and a third researcher (LTC) resolved discrepancies between the other two researchers during the screening processes.

### 2.4. Data Extraction and Synthesis

In order to make data synthesis more objective, we created three tables for the information to be collected from each included article, before starting the data extraction. These tables were elaborated by consensus of all the researchers and were based on the *Cochrane Handbook* recommendations [22]. The first table included items related to the main characteristics of the studies: author/year, study design, sample/country, participants, intervention/comparator, assessment and main study variables [24]. The second table included items related to the specific characteristics of the play-based interventions: author/year, participants and diagnosis, intervention, intervention duration, number of sessions, intervention manager and main results. The last table included items related to the risk of bias of the included articles: author/year, main limitations, funding sources and declarations of interest [25]. Two researchers (LCLN and LMCG) were in charge of data extraction, and a third researcher (LTC) was responsible for resolving possible discrepancies during data extraction. We present a narrative description of the results in this scoping review and we have used tables and figures wherever possible.

### 2.5. Quality Assessment

We did not assess the quality of the included articles as it is not a mandatory process in scoping reviews [26,27,28]. However, as recommended in the *Cochrane Handbook*, we have included a table on the risk of bias in each of the included articles to alert readers to aspects closely related to low quality such as limitations, funding sources and declarations of interest [22]. In addition, we have added a subsection on results providing a summary of the main limitations reported in the included studies, which can also be an indicator of the quality of the studies.

## 3. Results

**Figure 1:**
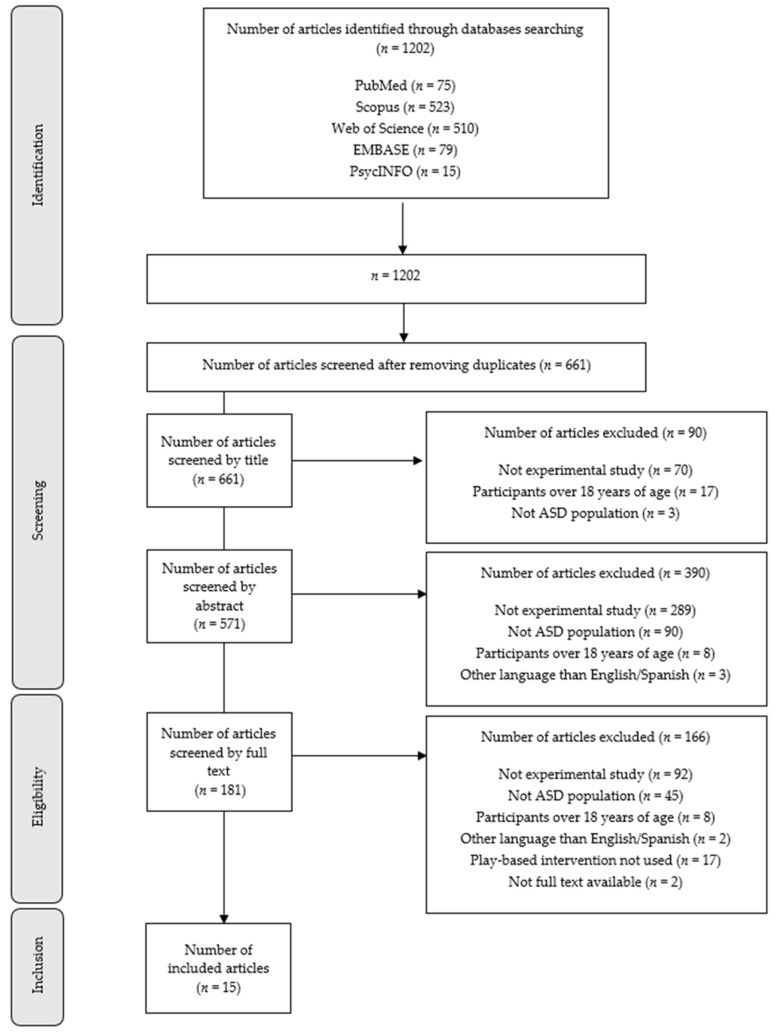
Flowchart of the study selection process.

We obtained a total of 1202 articles from the literature searches conducted in the five databases. After eliminating duplicates, we screened 661 articles by title, then the remaining 571 were screened by abstract, leaving 181 which were screened by full text. Of these 181 articles, only 15 met the inclusion criteria and were included in this scoping review (Figure 1).

### 3.1. Participants and Sample

The included articles were conducted in the following countries: Australia (*n* = 6) [29,30,31,32,33,34], the USA (*n* = 3) [35,36,37], France (*n* = 1) [38], Italy (*n* = 1) [39], Spain (*n* = 1) [40], China (*n* = 1) [41], Canada (*n* = 1) [42] and India (*n* = 1) [43] (Table 2). The sample size of the included studies ranged from 10 [31] to 76 participants [33]. The age of the participants ranged from 1 to 14 years in all studies, except in one in which the age ranged from 2 to 33 years [39]. However, the average age of the participants was below 18 years in all the included studies.

In all the included studies the study population consisted of children and adolescents with ASD, but in two of them the diagnoses were specifically Rett syndrome [39] and Asperger syndrome [33]. In some studies, in addition to ASD, the study population was made up of typically developing children and adolescents (*n* = 4) [30,32,33,36] or by children and adolescents with other disorders, such as Down’s syndrome (*n* = 1) [43] (Table 2).

### 3.2. Design of Included Studies

The included studies were randomized controlled trials (*n* = 7) [29,32,33,34,35,39,40], non-randomized controlled trials (*n* = 7) [30,31,36,37,38,41,43] and a pilot study (*n* = 1) [42] (Table 2). Eight of the studies did not have a control group (*n* = 8) [29,31,32,36,37,40,41,43], while seven did (*n* = 7) [30,33,34,35,38,39,42]).

Most of the control groups were characterized by not having received any intervention (*n* = 4) [32,38,39,42], or by having received either: the same intervention as the intervention group but with a different session duration (*n* = 1) [35], the same intervention as the intervention group but with different intervention duration (*n* = 1) [30] or a different intervention to the intervention group (*n* = 1) [34] (Table 2).

### 3.3. Study Variables and Measurement Instruments

Significantly, few articles (*n* = 3) used playing as a study outcome. In this sense, pretend play was assessed using the Child-Initiated Pretend Play Assessment (ChIPPA) [29], interactive peer play skills were assessed using the Penn Interactive Peer Play Scale (PIPPS) [29], playtime interaction was assessed by the Peer Interaction Paradigm (PIP) [35] and behavior during play was assessed by video analysis [40] (Table 2).

Behavior was the most studied outcome among the included studies (*n* = 7) (Table 2). Usually, only one questionnaire or battery was used per article to assess behavior. The evaluation tools were: the Conners Comprehensive Behavior Rating Scales (CCBRS) [31,33], the Early Head Start (EHS) 24-Month 3-Bag Scales [36], the Functional Emotional Assessment Scale (FEAS) [37], the Eyberg Child Behavior Inventory-Parent (ECBI) [34] and the Achenbach System of Empirically Based Assessment (ASEBA) [40]. However, one included article [42] used the following five questionnaires to assess behavior: the Behavior Rating Inventory of Executive Function (BRIEF), the Conners Rating Scale-Short Version (CRS-3), the Behavioral and Emotional Rating Scale 2nd edition (BERS-2), the Social Skills Rating System (SSRS) and the Gilliam Autism Rating Scale 2nd edition (GARS-2).

Language was the second most studied outcome among the included studies (*n* = 5) (Table 2). Four studies assessed language as a global function, using the following tools: The Preschool Language Scale 4th edition (PLS-4) [29], the Children’s Communication Checklist 2nd edition (CCC-2) [31,32], the Expressive Vocabulary Test 2nd Edition (EVT-2) and the Elaborated Phrases and Sentences subtest of the Test for Auditory Comprehension of Language 4th Edition (TACL-4) [32,33]. Another four studies assessed specific aspects of language such as literacy skills using the SEMA-TIC [38], French reading skills using the Alouette Reading Test [38], pragmatic language using the Pragmatics Observational Measure (POM) [31,33], reception and expression of prosody using the Profiling Elements of Prosody in Speech Communication (PEPS-C) [31], language performance during social interaction using Pragmatics Observational Measure 2 (POM-2) and Social Emotional Evaluation (SEE) [32,33]. Another study assessed language development disorder using the Children’s Communication Checklist 2nd edition (CCC-2) [33].

Social skills (*n* = 4) and intellectual skills (*n* = 4) were the third most studied outcome among the included studies. Social skills assessment was performed using the Social Emotional Evaluation (SEE) [31], the Social Skills Rating System (SSRS), the Aberrant Behavior Checklist (ABC) [40], the Conners Comprehensive Behavior Rating Scales (CCBRS) [32] and the Social Skills Questionnaire (SSQ) [34]. Intellectual skills assessment was performed using the Wechsler Intelligence Scale for Children 4th edition (WISC-IV) [40], the Conners Comprehensive Behavior Rating Scales (CCBRS) [32,33], the Oral Reading Fluency (ORF) [42], the Curriculum Based Measure [42] and the Woodcock Johnson III-Math Fluency (WJ-III) [42] tests.

Other study outcomes on aspects such as attention [39], working memory [43], executive functions, stress [35] and gross motor skills [30] were assessed in isolation in some studies using a variety of questionnaires (Table 2).

### 3.4. Play-Based Interventions

The duration of interventions used in the included studies was 11 weeks, except for one that lasted 32–48 weeks [37] and one for which the duration is unknown [36] (Table 2). The sessions usually took place once a week (*n* = 8) [31,32,33,34,35,38,40,41], although in some studies they were carried out twice [29,36], three [30,42] or five times a week [39]. In one article the number of sessions varied between two and five times a week [43], and in another article the number of sessions was not reported [37].

Sessions in most studies (*n* = 11) lasted from 30 min to 1 h [29,30,31,32,33,34,39,40,41,42,43], except in two studies in which they lasted 4 h [35,38] and in another in which they lasted 10 min [36]. The duration of each session was not stated in one article [37] (Table 3).

We classified the play-based interventions used in the included studies into three main groups: play-based interventions using new technologies, play-based interventions using symbolic play and play-based interventions using official techniques.

### 3.5. Play-Based Interventions Using New Technologies

In seven of the included articles, a play-based intervention using new technologies was carried out (Table 3). All these articles were published during the past five years, specifically in 2017 (*n* = 2) [30,38], 2019 (*n* = 2) [39,40] and 2021 (*n* = 3) [34,42,43]. The technologies used in these articles were computer games (*n* = 3) [34,38,42], digital games with devices [30,39,40] and smartphone games (*n* = 1) [43].

Three different computer games were used as an intervention strategy. Firstly, Serret et al. [38] used the SEMA-TIC which is a computerized game based on non-verbal cognitive skills and consists of ten sets of ten games and a dictionary in order to teach the player how to use a computer mouse. In this study, participants were aged from 6 to 11 years and they underwent 23 weeks of intervention with one weekly 4 h session. The intervention was conducted by speech therapists, psychologists, teachers and the children’s parents. Secondly, Beaumont et al. [34] used the Secret Agents Society (SAS), a computer resource for people with communication difficulties with the aim of improving face-to-face communication by overcoming different “game missions”, which facilitates training in social skills. In this study, participants were aged from 4 to 6 years and they underwent 10 weeks of intervention with one weekly 30 min session. The intervention was conducted by occupational therapists and the children’s parents. Thirdly, Macoun et al. [42] developed and used the Caribbean Quest, a cognitive training program based on virtual games with the aim of improving attention and executive function skills through repetitive and hierarchical games based on neuroplasticity, using cognitive strategies to generalize the use of these abilities to the real world. In this study, participants were aged from 6 to 12 years and they underwent 8 weeks of intervention with three weekly 30 min sessions. The intervention was conducted by teachers.

Three other interventions were carried out using virtual games through digital devices such as the Xbox Kinect device (Microsoft, Redmond, WA, USA) and an eye-tracker. Edwards et al. [30] carried out an intervention using three sports video games through the Xbox Kinect device. This device has a sensor which allows full body movements with a free range of motion, and was therefore used to improve gross motor abilities and the control of objects. In this study, participants were aged from 6 to 10 years and they underwent 2 weeks of intervention with three weekly 45–60 min sessions. The intervention was conducted by the children’s parents. The Xbox Kinect device was also used by Mairena et al. [40] but this time with the Pico’s Adventure game, in which the child must help an alien that has just landed on Earth to address social initiation behaviors. In this study, participants were aged from 4 to 6 years and they underwent 4 weeks of intervention with one weekly 60 min session. The intervention was conducted by psychologists. The last devices used in the included studies was the Tobii I-Series, an eye-tracker, which is a device that registers eye movements and sends them to specific software to generate gaze data. Fabio et al. [39] used this device with the Sensory eye FX program, which consisted of a set of thirty applications to analyze attention and motivation during digital games play. In this study, participants were aged from 2 to 33 years and they underwent 16 weeks of intervention with five weekly 30 min sessions. The intervention was conducted by the researchers.

The smartphone was the last technology used in play-based interventions. Wagle et al. [43] used five different smartphone games for mobility management training which contained sensorimotor and behavioral components. In this study, participants were aged from 6 to 13 years and they underwent 4 weeks of intervention with 2 to 5 weekly 30 min sessions. The intervention was conducted by teachers.

### 3.6. Play-Based Interventions Using Symbolic Play

In six of the included articles, a play-based intervention using symbolic play was carried out (Table 3). These articles were published between 2012 and 2020; specifically, in 2012 (*n* = 1) [29], 2017 (*n* = 1) [36], 2019 (*n* = 3) [31,32,33] and 2020 (*n* = 1) [41].

Stagnitti et al. [29] carried out the group-based intervention Learn to Play Program, which consisted of transport, construction and household games, as well as caring for dolls, in order to develop play and simulation skills. In these games, the child was in charge of taking photographs while playing, in order to talk about them later and, in this way, work on language skills. In this study, participants were aged from 5 to 6 years and they underwent 24 weeks of intervention with two weekly 60 min sessions. The intervention was conducted by teachers, speech and language therapists and occupational therapists.

MacDonald et al. [36] carried out a play-based intervention in two different scenarios. The first was based on social play, in which cause–effect toys, construction, cars, miniatures and imaginative play were used. The second was based on motor abilities in which stairs, mats, slides, see-saws and balls were used in an open space. Authors instructed children to “play together as usual”, and then they were recorded. In this study, participants were aged from 2 to 7 years and they underwent two days of intervention with one daily 10 min session in each scenario. The intervention was conducted by the researchers.

Parsons et al. carried out three studies in 2019 [31,32,33] in which a pragmatic language intervention based on play was delivered with different elements such as sandboxes, animal toys, dolls clothes, board and card games or building blocks. In this intervention, children were asked to play in pairs, putting children with ASD together with typically developing children. The sessions were recorded and the participants rated the game as “green” or “red” according to whether they thought it was favorable for both members of the pair or unfavorable for one or both, respectively. In these studies, participants were aged from 6 to 12 years and they underwent 10 weeks of intervention with one weekly 50–65 min session. The intervention was conducted by speech therapists, occupational therapists and parents.

The last study in which play-based intervention using symbolic play was used was a study carried out by Fu et al. [41]. They used a play-based communication and behavioral intervention including various elements such as cause–effect toys, stories, balls, card games, dolls or building blocks. In this intervention, each week occupational therapists taught a new technique to mothers of children with ASD to practice with their 1–2-year-old children. In this study, participants were aged from 1 to 2 years and they underwent 12 weeks of intervention with one weekly 60 min session. The intervention was conducted by occupational therapists and the children’s mothers.

### 3.7. Play-Based Interventions Using Official Techniques

In two of the included articles, published in 2007 [37] and in 2017 [35], a play-based intervention using official or registered techniques was carried out (Table 3).

Corbett et al. [35] used a program called SENSE Theatre^®®^ which involved observing, interpreting and articulating thoughts and feelings through improvisation, role play, scripted interaction and acting. This program is aimed at improving social skills in children and adolescents with ASD through theatrical techniques delivered in a supportive and peer-mediated intervention. In this study, participants were aged from 8 to 14 years and they underwent 10 weeks of intervention with one weekly 4 h session. The intervention was conducted by clinical researchers.

Solomon et al. [37] carried out the PLAY Project Home Consultation (PPHC) program, which is a home-based program using the DIR^®®^/Floortime approach. This home-based counseling program was created to improve parent–child interaction by training parents of children with ASD in skills and techniques to use during play. A professional visited the families each month to give advice on the play sessions, which were videotaped. In this study, participants were aged from 1.5 to 6 years and they underwent 32–48 weeks of intervention in which parents were encouraged to play 15 h per week with their children. The intervention was conducted by social workers, recreational therapists, home-based consultants and the parents of children with ASD [37].

### 3.8. Main Results of the Included Studies

All studies showed improvement in most of their outcomes (Table 3). However, three of the included studies reported negative results [33,36,40] such as less parental involvement during play in the group of children with ASD compared to typically developing children [36], more maladaptive behaviors during video game intervention (intervention group) compared to free play (control group) [40] and worse post-intervention scores on discourse coherence in children with ASD [33].

In nine of the included studies, no significant differences between groups or pre-post intervention were found [30,32,33,35,36,37,41,42,43]. In one of these studies, there were no differences between the intervention and control groups in anxiety and cortisol levels [35]. In the remaining eight studies, there were no differences before and after intervention in different outcomes such as object control [30], child’s negativity towards the parents during social play [36], effectiveness of parents’ involvement with their children [37], language performance during social play [32], receptive syntax and expressive language [33], self-efficacy [41], divided and sustained attention [42] and working memory [43].

### 3.9. Main Limitations of the Included Studies

The most frequent limitation was the small sample size, reported in eight of the studies [30,32,33,35,36,40,42,43]. Five studies did not take into account variables that could have affected the results [29,30,32,33,35]. The intervention duration was too short in five studies [30,33,36,40,43] and in another five studies, authors reported a low generalizability of the results [30,34,38,39,41]. Less frequently mentioned limitations were: a lack of follow-up (*n* = 4) [33,40,41,42], the absence of a control group (*n* = 3) [37,41,42], non-randomization (*n* = 2) [38,42], non-blinding studies (*n* = 2) [40,42], subjective assessment (*n* = 2) [34,35], possibly overestimated data (*n* = 1) [35], low return rates for post-test evaluation (*n* = 1) [42], limitation of setting (*n* = 1) [36], results which were difficult to explain (*n* = 1) [37], and questionable reliability and validity of diagnosis and treatment (n = 1) [34].

### 3.10. Indicators of Bias in the Included Articles

In relation to funding, two articles reported having received private funding (*n* = 2) [30,41], another two received public funding (*n* = 2) [38,42], one was self-funded (*n* = 1) [34] and one received no financial support (*n* = 1) [39]. The remaining articles did not provide information regarding funding [29,31,32,33,35,36,37,40,43] (Table 4).

Only one of the included articles reported a conflict of interest, stating that one of the authors belonged to the company developing the program and received financial benefits, while the other authors reported no conflict of interest [34]. The remaining articles (*n* = 8) either declared no conflict of interest [30,31,32,33,38,39,41,42,43] or did not include information on this subject (*n* = 6) [29,33,35,36,37,40] (Table 4).

## 4. Discussion

Play-based interventions for children and adolescents with ASD were mainly characterized by the following characteristics: a duration of approximately 11 weeks, one weekly session of 30–60 min, conducted at school or at the participants’ home, and were delivered by the parents of children with ASD or teachers. In addition, most play-based interventions included new technologies or symbolic play, and rarely included registered or official techniques.

Most of the articles included in this review have been published in the past ten years and the majority of studies have been carried out in Australia. Rice et al. attributes this increase in research during the past few years to several causes, such as increased awareness of ASD, changes in diagnostic criteria of ASD and the need to know the risk factors associated with this disorder in more depth [44]. The incidence of publications on ASD has increased in recent years, as has the incidence of the disorder itself, especially in underdeveloped and developing countries [6]. In this sense, one of the continents with the highest prevalence of ASD is Oceania, where 697 out of every 100,000 children receive this diagnosis [7]. This fact could explain why Australia was one of the countries in which most studies were carried out.

The participants in the included studies were mostly male and around 6 years old. ASD is known to be more prevalent in boys than in girls [45,46]. Duvekot et al. try to explain this fact, stating that boys tend to show more symptoms that are characteristic of ASD such as repetitive and restricted behaviors (e.g., playing with dinosaurs or means of transport), while girls show more emotion-related symptoms and behavioral problems [47] and have a greater tendency to disguise their symptoms or difficulties [48]. As a result, ASD in girls may be commonly mistaken for other pathologies or mental health problems [47,48,49]. All these reasons can make the diagnosis of ASD in girls more difficult, and can, in some way, contribute to the low prevalence in girls. Finally, the fact that the most common age of the participants among the included studies was 6 years could be because ASD diagnosis is based on a series of symptoms which stand out more in a school setting, and therefore coincide with the beginning of primary school, at around 6 years [50].

The most used strategy in play-based interventions studied in the included articles was the use of new technologies. Moreover, it should be noted that all the play-based interventions using symbolic play included some elements that could be considered technological, such as digital photographs or video cameras. The wide use of technology may be due to the fact that children and adolescents currently belong to the digital era, i.e., technology is on the rise and is part of their daily lives [51] and, therefore, the use of new technologies can motivate them to follow the intervention. Another reason that can explain the frequent use of new technologies in play-based interventions is that nowadays many technological devices are easy to acquire and are commonly found in private homes, schools and clinics, especially computers, tablets and/or smartphones, increasing their accessibility [52]. In addition, children and adolescents with ASD present a clear preference for games related to new technologies [53], and therefore, their use in interventions may help professionals to increase these children and adolescents’ motivation and interest in rehabilitation, which may result in better adherence to treatment [52,54].

Play-based interventions were mostly delivered by parents, and to a lesser extent, by teachers. On the one hand, this result may be justified by the results shown by Althoff et al. in their recently published systematic review [55]. They showed that parent-mediated interventions for children and adolescents with ASD lead to an improvement in several outcomes related to children, such as attention, language, non-verbal communication, social communication, interaction, play, adaptive functioning and ASD symptoms [55]. On the other hand, teachers are one of the groups of professionals that spend most time with children and, therefore, are in a position to identify difficulties in children with ASD earlier, especially difficulty in social play, which is more easily detected in a school setting [55]. This is why training and involving teachers in play-based interventions is important for the inclusion of children with ASD in the school environment, one of the most important settings in a child’s life [56,57].

Children and adolescents with ASD also present deficits in social cognition (SC) [58]. Animal studies try to explain SC deficits in people with ASD by anatomical differences in some brain areas related to social competence [59]. SC is defined as a variety of cognitive skills necessary to recognize and use socially relevant information to respond adequately in social situations [60]. In this sense, some authors have described that play is essential for increasing the development of social skills in general [61], thanks to improvements in aspects of SC such as joint attention, social referencing and mentalization due to the development of problem-solving abilities [62,63]. Thus, play-based interventions can be an optimal treatment option to increase SC and should be complemented with a specific and adequate evaluation of SC [64].

Finally, we would like to point out that the present scoping review can be considered as a complementary document to the systematic review carried out by Dijkstra-de Neijs et al. in 2021 [21]. In this systematic review, the authors evaluate the effectiveness of play-based interventions for children and adolescents with ASD, while in our scoping review we describe the characteristics of play-based interventions for children and adolescents with ASD. There is a discrepancy in the number of articles reviewed between the two reviews, possibly because Dijkstra-de Neijs et al. used the Google Scholar database in their literature searches and we did not. This systematic review showed that play-based interventions mainly improve primary dimensions of ASD, specifically communication and social interaction. To these results, we can add that play-based interventions to improve social interaction seem to be characterized by the use of new technologies (Kinect Pico’s Adventure and the Secret Agent Society computer game) and delivered once a week, while play-based interventions to improve communication seem to be child-directed free play with therapists and peers, delivered once a week. However, it should be noted that these characteristics have been based on a small number of articles and more research is needed to draw conclusions.

The present scoping review has some limitations. First, as in most review articles, we cannot rule out publication bias, which leads to under-representation of null results of interventions in published articles. However, in the present review we have included several articles in which we found null results. The second limitation is the selection bias, also present in most review articles. We may have been increased it by excluding articles published in different languages other than Spanish or English. However, we have included articles written in English, the language in which the majority of the articles are written. This bias may also have been magnified by only including articles with full text available because we may have overlooked some potentially relevant articles for this scoping review. Third, it was difficult for us to establish the search strategy because the classification of ASD has changed in recent years, resulting in a lack of agreement about which disorders it includes. Thus, we decided to include all the following ASD disorders shown in the American Psychiatric Association’s (APA) *DSM-IV*, 2002 [65] in our search strategy: classic autistic disorder, pervasive developmental disorders, Rett syndrome, childhood disintegrative disorder and Asperger syndrome. As a result, studies analyzing other related disorders present in the ICD-10 may have been overlooked. Fourth, only experimental studies were included, so there may be some biases associated with this type of design, such as results based on a small sample size or a low representative sample. Fifth, the quality of the articles included was not assessed and, although this is not a mandatory requirement in scoping reviews, this may have led to the inclusion of low-quality articles. However, we have included a description of the main limitations of each included article in our results section and we have also collected and shown different issues closely related to the studies’ quality, such as funding and conflict of interest, in Table 4. With all this information, we wanted to make readers aware of the different quality indicators of each article so they could be careful when interpreting the results shown in this scoping review.

We can highlight some strengths such as the fact that no other reviews have been found that deal with the same study objective as ours. However, the greatest strength of any scoping review is the identification of knowledge gaps [66] which require new research and more scientific evidence. In this regard, we can highlight that: (1) Europe is very poorly represented in studies on play-based interventions for children and adolescents with ASD; (2) the structure of play-based interventions is unclear and therefore difficult to replicate.

## 5. Conclusions

Play-based interventions can be classified into those using new technologies, symbolic play or official techniques, but the most used and current are those using new technologies. These interventions were mainly delivered by parents or teachers of children and adolescents with ASD. They lasted approximately 11 weeks and comprised one weekly session, although this varied between studies. Further studies are needed to create play-based intervention protocols which can be implemented in clinical practice with children and adolescents with ASD, thus promoting evidence-based interventions in this field.

## Figures and Tables

**Table 1:** Databases and search strategies used.

Databases		Search Strategy 9 November 2021
PUBMED	#1	(ASD OR autism OR autistic OR asperger OR rett OR pervasive OR disintegrative)
	#2	(“game-based” OR “play-based”)
	#3	#1 AND #2
SCOPUS	#1	TITLE-ABS-KEY ((asd OR autism OR autistic OR asperger OR rett OR pervasive OR disintegrative))
	#2	TITLE-ABS-KEY ((“game-based” OR “play-based”))
	#3	TITLE-ABS-KEY ((asd OR autism OR autistic OR asperger OR rett OR pervasive OR disintegrative) AND (“game-based” OR “play-based”))
EMBASE	#1	‘asd’/exp OR asd OR ‘autism’/exp OR autism OR autistic OR asperger OR rett OR pervasive OR disintegrative
	#2	‘game-based’ OR ‘play-based’
	#3	#1 AND #2
Web of Science	#1	(ASD OR autism OR autistic OR asperger OR rett OR pervasive OR disintegrative)
	#2	(“game-based” OR “play-based”)
	#3	(ASD OR autism OR autistic OR asperger OR rett OR pervasive OR disintegrative) AND (“game-based” OR “play-based”)
PsycINFO	#1	(ASD OR autism OR autistic OR asperger OR Rett OR pervasive OR disintegrative)
	#2	(“game-based” OR “play-based”)
	#3	(ASD OR autism OR autistic OR asperger OR rett OR pervasive OR disintegrative) AND (“game-based” OR “play-based”)

**Table 2:** Characteristics of the studies included in this scoping review.

Author, Year	Design	Sample (*n*), Country	Participants	Intervention/Comparator	Evaluation	Study Outcomes
Solomon et al. (2007) [37]	nRCT	74, USA Loss to follow-up (*n* = 6)	68 children with ASD, Asperger or PDD-NOS-parent dyads (51 boys and 17 girls) Age: 1′5–6 years	The PLAY Project Home Consultation program/ NA	Pre- and post-evaluation	Behavior and development assessed by FEAS
Stagnitti et al. (2012) [29]	RCT	19, Australia Loss to follow-up (*n* = 0)	10 children with ASD, 9 children with other diagnosis (11 boys and 8 girls) Age: 5–6 years	The Learn to Play Program/NA	Pre- and post-evaluation	Pretend play assessed by ChIPPA Language assessed by PLS-4 Interactive peer play competencies assessed by PIPPS
Corbett et al. (2017) [35]	RCT	30, USA Loss to follow-up (*n* = 6)	IG: 17 children with ASD (13 boys and 4 girls) CG: 13 children with ASD (11 boys and 2 girls) Age: 8–14 years	SENSE Theatre^®®^—sessions/summer camp	Pre- and post- evaluation	Anxiety and stress assessed by STAI-C and Cortisol levels Playground interaction assessed by PIP
Edwards et al. (2016) [30]	nRCT	30, Australia Loss to follow-up (*n* = 0)	IG: 11 children with ASD (8 boys and 3 girls) CG: 19 children with TD (10 boys and 9 girls) Age: 6–10 years	AVG (Xbox Kinect: sportive games)/NA	Pre- and post- evaluation	Gross motor abilities assessed by TGMD-3 Perceived competence in object control skills assessed by PMSC
MacDonald et al. (2017) [36]	nRCT	18, USA Loss to follow-up (*n* = 0)	9 children with ASD, 9 children with TD (15 boys and 3 girls) Age: 2–7 years	Social play setting/motor behavior-based setting.	Pre- and post- evaluation	Cognitive development assessed by MSEL Child behaviors assessed by EHS
Serret et al. (2017) [38]	nRCT	30, France Loss to follow-up (*n* = 5)	IG: 12 children with ASD (11 boys and 1 girl) CG: 13 children with ASD (10 boys and 3 girls) Age: 6–11 years	Training group with SEMA-TIC game/control group	Pre- and post- evaluation	Literacy skills assessed by the SEMA-TIC French reading skills assessed by the Alouette Reading Test
Fabio et al. (2019) [39]	RCT	52, Italy Loss to follow-up (*n* = 0)	IG: 30 girls with RTT CG: 22 girls with RTT Age: 2–33 years	Eye-gaze digital games/control group	Pre- and post-evaluation	Rett syndrome assessed by RARS Attention, motivation and functionality assessed by different software scores
Mairena et al. (2019) [40]	RCT	20, Spain Loss to follow-up (*n* = 5)	15 children with ASD (15 boys) Age: 4–6 years	Free play with toys+ Kinect-based game Pico’s Adventure/Kinect-based game Pico’s Adventure + free play with toys	Pre- and post- evaluation and observation during sessions	Intellectual abilities assessed by WISC-IV Conduct during playing assessed by video analysis Maladaptive functioning and psychiatric symptoms assessed by ASEBA Effectiveness of treatments assessed by ATEC Social behaviors assessed by SSRS and ABC
Parsons et al. (2019) [31]	nRCT	10, Australia Loss to follow-up (*n* = 0)	10 children with ASD-TD peer–parents trio (14 boys and 6 girls) Age: 6–12 years	Play-based and peer-mediated pragmatic language intervention/NA	Pre-, post-evaluation and 2-months follow-up	Pragmatic language assessed by POM Social skills assessed by SEE Reception and expression of prosody assessed by PEPS-C General language assessed by CCC-2 Behaviour and emotions assessed by CCBRS
Parsons et al. (2019) [32]	RCT	71, Australia Loss to follow-up (*n* = 9)	IG: 28 children with ASD-TD peer–parents trio (38 boys, 15 girls) CG: 34 children with ASD-TD peer–parents trio (48 boys, 21 girls) Age: 6–11 years	Play-based and peer-mediated pragmatic language intervention/control group	Pre-, post- and 3-month follow-up evaluation	Language performance during social play assessed by POM-2 and SEE Language assessed by EVT-2, the Elaborated Phrases and Sentences subtest of TACL-4 and CCC-2 Academic and social problems assessed by CCBRS
Parsons et al. (2019) [33]	RCT	76, Australia Loss to follow-up (*n* = 16)	60 children with Asperger-TD peer–parents trio (51 boys and 9 girls) Age: 6–11 years	Play-based and peer-mediated pragmatic language intervention/control group	Pre- and post- evaluation	Pragmatic language assessed by POM-2 Language assessed by EVT-2, TACL-4 and SEE Behavioral and academic abilities assessed by CCBRS Developmental language disorder assessed by CCC-2
Fu et al. (2020) [41]	nRCT	70, China Loss to follow-up (*n* = 0)	70 children with ASD-mother dyads (55 boys and 15 girls) Age: 1–2 years	Play-based communication and behavior intervention (PCBI)/NA	Pre- and post-evaluation	Efficacy of PCBI assessed by ATEC Developmental quotient assessed by the Gesell Developmental Schedules
Beaumont et al. (2021) [34]	RCT	70, Australia Loss to follow-up (*n* = 6)	IG: 31 children with ASD-parent dyads (30 boys and 1 girl) CG: 33 children with ASD-parent dyads (30 boys and 3 girls) Age: 7–12 years	Face-to-face Secret Agent Society (SAS) multimedia-based group intervention/caregiver-supported cognitive skills training game (CIA)	Pre-, post- evaluation and 6-weeks follow-up	Social skills assessed by SSQ Emotion competence assessed by ERSSQ Anxiety assessed by SCAS-P Behavior assessed by ECBI
Macoun et al. (2021) [42]	Pilot	23, Canada Loss to follow-up (*n* = 3)	IG: 11 children with ASD (9 boys and 2 girls) CG: 9 children with ASD (8 boys and 1 girl) Age: 6–12 years	Game-based cognitive training program (Caribbean Quest)/control group	Pre- and post-evaluation	Attention and EF assessed by KiTAP and WISC-IV Academic achievement assessed by ORF, curriculum based measure and WJ-III Behavioral ratings assessed by BRIEF, CRS-3, BERS-2, SSRS and GARS-2
Wagle et al. (2021) [43]	nRCT	19, India Loss to follow-up (*n* = 5)	14 children with ASD or DS (11 boys and 3 girls) Age: 6–13 years	Smartphone-based games/NA	Pre- and post-evaluation	WM assessed by the standard in-person Corsi-block tapping task Intervention effectiveness assessed by ATEC

ABC: Aberrant Behavior Checklist; ASD: autism spectrum disorder; ASEBA: Achenbach System of Empirically Based Assessment; ATEC: Autism Treatment Evaluation Checklist; AVG: active video games; BERS-2: Behavioral and Emotional Rating Scale, second edition; BRIEF: Behavior Rating Inventory of Executive Function; CCBRS: Conners Comprehensive Behaviour Rating Scales; CCC-2: Children’s Communication Checklist, Second Edition; CG: control group; ChIPPA: Child-Initiated Pretend Play Assessment; CIA: Central Intelligence Agency; Cortisol: levels of cortisol; CQ: The Caribbean Quest; CRS-3: Conners Rating Scale-Short Version; DD: developmental delay; DGBL: digital game-based learning; DIR: developmental, individual differences, relationship-based; DS: Down’s syndrome with symptoms of ASD; ECBI: Eyberg Child Behavior Inventory-Parent; EF: executive function; EHS: Early Head Start (EHS) 24-Month 3-Bag Scales; ERSSQ: Emotion Regulation and Social Skills Questionnaire; EVT-2: Expressive Vocabulary Test, Second Edition; FASD: fetal alcohol spectrum disorder; FEAS: Functional Emotional Assessment Scale; G1: intervention first; G2: waitlist first; GARS-2: Gilliam Autism Rating Scale; GSES: General Self-Efficacy Scale; I1: intervention group 1; I2: intervention group 2; IG: intervention group; IY: The Incredible Years; KiTAP: Test of Attentional Performance for Children; Loss: loss to follow-up; MSEL: Mullen Scales of Early Learning; NA: not applicable; nRCT: no randomized control trial; PCBI: play-based communication and behavior intervention; ORF: oral reading fluency; PDD-NOS: pervasive developmental disorder not otherwise specified; PEPS-C: Profiling Elements of Prosody in Speech Communication; Pilot: Pilot study of a RCT; PIP: Peer Interaction Paradigm; PIPPS: Penn Interactive Peer Play Scale; PLS-4: Preschool Language Scale, Fourth Edition; PMSC: Pictorial Scale of Perceived Movement Skill Competence for Young Children; POM/POM-2: Pragmatics Observational Measure, First/Second Edition; PPHC: PLAY Project Home Consultation; PSI-SF: Parenting Stress Index Short Form; RARS: Rett Assessment Rating Scale; RCT: randomized control trial; RTT: Rett syndrome; SAS: Secret Agent Society; SCAS-P: Spence Children’s Anxiety Scale-Parent (SCAS-P); SEE: Social Emotional Evaluation; SSQ: Social Skills Questionnaire; SSRS: Social Skills Rating System; STAI-C: State-Trait-Anxiety Inventory for Children; TACL-4: Test for Auditory Comprehension of Language, Fourth Edition; TD: typically developing; TGMD-3: Test of Gross Motor Development, Third Edition; USA: United States of America; WISC-IV: Wechsler Intelligence Scale for Children, fourth edition; WJ-III: Woodcock Johnson III-Math Fluency.

**Table 3:** Characteristics of the interventions performed in the included studies.

Author, Year	Participants and Diagnosis	Interventions	Duration (w)	Sessions	Intervention Managers	Main Results
Solomon et al. (2007) [37]	74, ASD, PDD-NOS and Asperger	The PLAY Project Home Consultation program including the DIR/Floortime approach. Home consultants make monthly, 3–4 h visits to families’ homes to teach parents how to provide intensive, one-on-one, play-based intervention to their young children with ASD.	32–48	15 h/week (Session duration NS)	Social workers, recreational therapists, home consultants, parents	Increase in children’s functional development in post- vs. pre-intervention (*p* ≤ 0.0001) in IG No differences in FEAS parent’s score in IG parents in post- vs. pre-intervention
Stagnitti et al. (2012) [29]	19, ASD and DD	The Learn to Play Programme. A child-led play-based intervention to develop play and simulation skills to fulfil the role of “player”. One child takes pictures of the present game to work on language in the next game.	24	60 min, 2 times/week	Teachers, SP, OT	Increase in social interaction, social connection and language in post- vs. pre-intervention (*p* < 0.01)
Corbett et al. (2017) [35]	30, ASD	SENSE Theatre^®®^: observing, interpreting and articulating thoughts and feelings through actor development training and video models	10	240 min, 1 times/week	Clinical researchers	Decrease in anxiety levels in IG in post- vs. pre-intervention (*p* = 0.005) No difference between groups in cortisol levels.
Edwards et al. (2016) [30]	30, ASD and TD	Active video games using the Xbox Kinect. Children with ASD play three different sportive games which allow full body movements to interact with the game to develop object control skills.	2	45–60 min, 3 times/week	Parents	Increase in perception of object control skills in post vs. pre-intervention in ASD group (*p* = 0.044), but no evident improvement in IG vs. CG (*p* > 0.05)
MacDonald et al. (2017) [36]	18, ASD and TD	Two play sessions, each taking place in a distinctly different setting. The social-play-based setting consisted of common toys typically used in a natural or free-play setting such as toy cars or imaginative play with blocks. The motor behavior-based setting: situated in a large play space in addition to common equipment to encourage motor behavior, such as play on stairs or a tricycle. The sessions were recorded.	2 days	10 min, 1 times/day	Researchers	Increase in engagement in social play, in engagement in motor play (*p* = 0.005), in sustained attention (*p* = 0.001) and parent–child connectivity (*p* = 0.014) in IG vs. CG Decline in parental involvement in IG group vs. CG (*p* value NS)
Serret et al. (2017) [38]	30, ASD	SEMA-TIC game. Computerized game based on non-verbal cognitive skills and trial-and-error strategy in order to learn computer mouse use. It included 10 sets of 10 games such as word picture associations or logic games with words.	23	240 min, 1 times/week	SP, psychologists, teachers, parents	Increase in tracking and segmentation test in IG vs. CG (*p* ≤ 0.001) Increase in letter and single word reading in post- vs. pre-intervention (*p* ≤ 0.001) in IG
Fabio et al. (2019) [39]	52, RTT	Digital game-based learning using Tobii Series-I (eye tracker). They used five eye-gaze games: blank screen engagement, object scrolling, zone focusing, active scanning and controlled orientation.	16	30 min, 5 times/week	Researchers	Increase in attention and motivation in post vs. pre-intervention (*p* < 0.01) in IG No differences in functionality in post vs. pre-intervention (*p* value NS) in IG and CG
Mairena et al. (2019) [40]	20, ASD	Kinect Pico’s Adventure game. A full body interaction video game with social initiation missions. (1) Plays alone but interacts with Pico with gestures and vocalizations (to obtain food); (2) first plays alone, then with an adult (for Pico to fix his spaceship); (3) the child gives instructions to an adult (to free the ships of Pico’s friends); (4) plays with another child with ASD he does not know (to obtain what the inhabitants of Pico’s planet offer them).	4	60 min, 1 times/week	Psychologists	Increase in social initiation when the child with ASD played alone, with another child with ASD (*p* = 0.003) or with their parents (*p* = 0.014) during videogame vs. free play (*p* = 0.033) Increase in social behaviors when played with parents in free play vs. videogame (*p* = 0.048) Increase in maladaptive behaviors in videogame vs. free play (*p* value NS) Decreased repetitive actions in videogame vs. Free Play (*p* = 0.040)
Parsons et al. (2019) [31]	10, ASD	Peer-mediated, play-based pragmatic language intervention, which consisted of 30 min child-directed free play with therapist, video feedback play and home training (DVD watching and then parent–child play).	10	55–65 min, 1 times/week	SP, OT, parents	Increase in pragmatic language (*p* = 0.011) and high-level language (*p* = 0.035) in post- vs. pre-intervention in IG
Parsons et al. (2019) [32]	71, ASD	Peer-mediated, play-based pragmatic language intervention: paired play with TD and parent-mediated practice at home. (1) 15–20 min therapist-led video-feedback; (2) 20 min child-led play with therapist modeling; (3) 15 min therapist–parent discussion.	10	50–55 min, 1 times/week	SP, OT, parents	Increase in pragmatic language in post- vs. pre-intervention (*p* = 0.031) in IG Maintenance of effects for at least 3 months post-intervention in IG (*p* < 0.001–0.05) No difference between POM-2 scores in clinic and at home during follow-up in IG
Parsons et al. (2019) [33]	76, Asperger	Peer-mediated, play-based pragmatic language intervention, which consisted of 30 min child-directed free play with therapist and peers, video feedback play and home training. The play in the clinic was recorded and the images formed the video-feedback for the following week.	10	50 min, 1 times/week	SP, OT, parents	No increase in receptive syntax, expressive language and speech coherence in post- vs. pre-intervention (*p* value NS) Increase in non-verbal communication in IG (*p* value NS)
Fu et al. (2020) [41]	70, ASD	Play-based behavioral and communication intervention: 2.5 days training of play with children for mothers. In each session mothers learn one technique at home, with feedback from therapist; then the therapist performs a demonstration of play with child; and mother interacts with child, with guidance from therapist.	12	60 min, 1 times/week	OT, mothers	Increase in ABC score (*p* < 0.001) and ATEC score (*p* < 0.001) in post- vs. pre-intervention Increase in mother–child bond (*p* < 0.001) in post- vs. pre-intervention Decrease in mothers’ disconnection (*p* = 0.001) and stress (*p* = 0.004) in post- vs. pre-intervention No difference in child self-efficacy (*p* = 0.318) in post- vs. pre-intervention
Beaumont et al. (2021) [34]	70, ASD	Secret Agents Society. A computer resource for families with barriers to face-to-face interventions aimed at generalizing social skills. In this game, participants are asked to carried out “home missions” (daily skills practice tasks) for everyday life. Families received a weekly video coaching session for problem solving and 150 min webinar with tips on behaviour management.	10	30 min, 1 times/week	OT, parents	Increase in social skills (*p* = 0.0005) in IG vs. CG, maintained at follow-up (*p* < 0.0005) Increase in emotional competence in IG vs. CG (*p* = 0.0005), maintained at follow-up (*p* < 0.0005) Increase in social skills in both groups (*p* value NS)
Macoun et al. (2021) [42]	23, ASD	Caribbean Quest game-based cognitive training program. Five mini-virtual games to improve attention and executive function skills with repetitive and hierarchical exercises.	8	30 min, 3 times/week	Rural teachers	Increase in selective attention in IG vs. CG and in post- vs. pre-intervention (*p* < 0.05) No differences in divided and sustained attention (*p* value NS) Increase in visual–spatial management in IG vs. CG and in post- vs. pre-intervention (*p* < 0.01)
Wagle et al. (2021) [43]	19, ASD and DS	Smartphone games: five games (basket, train, piano, face and shape) for mobility management training with sensorimotor and behavioral components.	4	30 min, 2–5 times/week	Teachers	No difference in working memory in one month of training (*p* value NS) Positive correlation between game scores and working memory Increase in autistic symptoms during treatment, but not maintained (*p* value NS) No correlation between working memory and autistic symptoms

ABC: Aberrant Behavior Checklist; ASD: autism spectrum disorder; ATEC: Autism Treatment Evaluation Checklist; CG: control group; DD: developmental delay; DS: Down’s syndrome with symptoms of ASD; FEAS: Functional Emotional Assessment Scale; IG: intervention group; min: minute/s; NS: not stated; OT: occupational therapist; PDD-NOS: pervasive developmental disorder not otherwise specified; POM-2: Pragmatic Observational Measure; RTT: Rett syndrome; SP: speech pathologist; TD: typically developing.

**Table 4:** Items related to risk of bias of included studies.

Author, Year	Main Limitations	Funding Sources	Declarations of Interest
Solomon et al. (2007) [37]	Lack of a CG, the results cannot be easily explained	NS	NS
Stagnitti et al. (2012) [29]	Managers cannot be changed, effect of other activities not assessed	NS	NS
Corbett et al. (2017) [35]	Small sample size, inflated scores in the IG, only self-reported measures of anxiety; alexithymia not assessed	NS	NS
Edwards et al. (2016) [30]	Small sample size, low generalizability of the results, shorter intervention duration in ASD group, physical activity intensity during game play not recorded	Department of Education Victoria. Alfred Deakin Fellowship. Supported by internal university funding. This research did not receive any specific grant from funding agencies in the public, commercial or not-for-profit sectors	The authors declare no conflict of interest
MacDonald et al. (2017) [36]	Small sample size, short intervention duration, limited environment	NS	NS
Serret et al. (2017) [38]	Low generalizability of the results, lack of randomization	The Regional Health Agency Provence-Alpes-Côte-d’Azur (ARS PACA)	The authors declare no conflict of interest
Fabio et al. (2019) [39]	Low generalizability of the results	The authors declare no source of funding	The authors declare no conflict of interest
Mairena et al. (2019) [40]	Small sample size, short intervention duration non-blinding study, lack of follow-up	NS	NS
Parsons et al. (2019) [31]	Small sample size, in some cases, the partner was the brother	NS	The authors declare no conflict of interest
Parsons et al. (2019) [32]	Pragmatic language abilities of the playmates not evaluated	NS	The authors declare no conflict of interest
Parsons et al. (2019) [33]	Small sample size, short intervention duration, reliability of the developed algorithms not evaluated in the IG, lack of follow-up	NS	NS
Fu et al. (2020) [41]	Lack of a CG, only mothers included, only short-term analysis was carried out	The National Key Research and Development Program of China (2016YFC1306200), the National Natural Science Foundation of China (81771478), and the Special disease cohort Research Project of Nanjing Medical University (NMUC2018010A)	The authors declare no conflict of interest
Beaumont et al. (2021) [34]	Homogeneous group of parents, questionable reliability and validity of treatment fidelity, possible ratter bias (subjective assessment), low rate of return of teacher measures, questionable validity of diagnostic tests	Royalty payments on all program materials and practitioner training courses sold	Renae Beaumont is the Secret Agent Society Program developer and receives royalty payments on all program materials and practitioner training courses sold. All other authors of this paper declare that they have no conflict of interest
Macoun et al. (2021) [42]	Small sample size, lack of a CG, lack of randomization, non-blinding study, lack of follow-up, low return rates for post-test rating scales	Kids Brain Health Network (previously NeuroDevNet), member of the Networks of Centres of Excellence program of Canada	The authors declare no conflict of interest
Wagle et al. (2021) [43]	Small sample size, short intervention duration	NS	The authors declare no conflict of interest

## Data Availability

The data presented in this study are available on request from the corresponding author.
